# Effect of Re-Recycling on Rheology and Microstructure of Asphalt Binder

**DOI:** 10.3390/ma15196641

**Published:** 2022-09-24

**Authors:** Ruipu Chen, Hongzhou Zhu, Li Ou, Yanling Xu

**Affiliations:** 1 School of Civil Engineering, Chongqing Jiaotong University, Chongqing 400074, China; 2 National and Local Joint Engineering Laboratory of Transportation and Civil Engineering Materials, Chongqing Jiaotong University, Chongqing 400074, China

**Keywords:** re-recycled asphalt binder, microstructure, FTIR, AFM

## Abstract

Currently, aged recycled asphalt pavements have re-recycling demands, but the evolution mechanism of re-recycled asphalt binder properties is still unclear. Therefore, this study analyzes the rheological properties and microstructure of re-recycled asphalt by dynamic shear rheometer (DSR), bending beams rheometer (BBR), atomic force microscope (AFM), and Fourier transform infrared spectroscopy (FTIR). The macro performance results show that re-recycling improves high-temperature performance and reduces fatigue and low-temperature performance. In addition, the aged re-recycled asphalt’s ΔT_c_ ≤ −2.5 °C, has a risk of low-temperature cracking. The micro results show that the adhesion between asphalt and aggregate decreases as the recycling times increase; the re-recycled asphalt mixture has a greater adhesion cracking risk. Some macro–micro experimental results are correlated. Aging accelerates the decay of rheological properties of re-recycled asphalt by increasing the microscopic roughness and carbonyl index of re-recycled asphalt. It indicates that re-recycling reduces the aging resistance of asphalt. Furthermore, the properties of recycled asphalt are strongly correlated with aging functional groups, roughness, and surface energy; the microstructural changes significantly influence the rheology properties of asphalt.

## 1. Introduction

The rapid development of the social economy has led to generating a large amount of solid waste [1], such as waste asphalt mixture. The reclaimed asphalt pavement (RAP) has been used for more than 100 years since the first recycling by Warren Brothers in the United States in 1915. With the development of civil engineering technology, recycled asphalt pavement is widely used [2]. Using RAP can reduce engineering costs and greenhouse gas emissions [3,4]. However, some practical problems exist, such as poor crack resistance of recycled asphalt mixtures with a large amount of RAP [5]. Therefore, some hot central-plant recycling controls the amount of RAP at about 30% without a rejuvenating agent to maintain better performance and to reduce engineering costs [6].

The aged recycled pavements have re-recycling demands; re-recycling has obvious economic and environmental advantages. According to literature records, Yoshikane [7] first proposed the concept of multiple recycled asphalt pavements in 1995. Japan was also an early country to study multiple recycling [8], followed by China, the United Kingdom, and other countries [9,10]. However, there are few published reports on re-recycled asphalt, and the understanding of re-recycling is still limited. Owing to the complex composition of multiple recycled asphalt [11], the performance evolution of multiple recycled asphalt has not reached a consistent conclusion; in particular, multiple recycled asphalt contains about 30% aged asphalt. Hugener [12] and Lidia Derossi [13] found that the effect of multiple recycling on the rheological properties of asphalt can be ignored. However, there are different opinions. Koudelka [14] and Nie [15] found that the differences in rheological properties between neat and multiple recycled asphalt widen as the recycling times increase. Most of the current research mainly focused on the macroscopic scale. However, there is a lack of mechanism research on the microscopic scale [16]. Currently, many advanced microscopic methods are commonly used to analyze the microstructure of asphalt, such as atomic force microscopy (AFM) and fourier transform infrared spectroscopy (FTIR). AFM is often used to analyze the micromorphology and mechanical characteristics of asphalt [17], and FTIR is used to analyze the changes in chemical functional groups [12]. Therefore, this article investigates the actual performance evolution mechanism of re-recycled asphalt by micro (AFM, FTIR) and macro (dynamic shear rheometer (DSR), bending beams rheometer (BBR)) methods. The main body of this article contains the following topics:

(1) Evaluating the evolution of rheological properties of the re-recycled asphalt;
(2) Analyzing the microstructure of re-recycled asphalt binder by FTIR and AFM;
(3) Correlating macro properties and microstructures of the asphalt.

## 2. Materials and Methods

### 2.1. Materials and Binder Preparation

**Figure 1:**
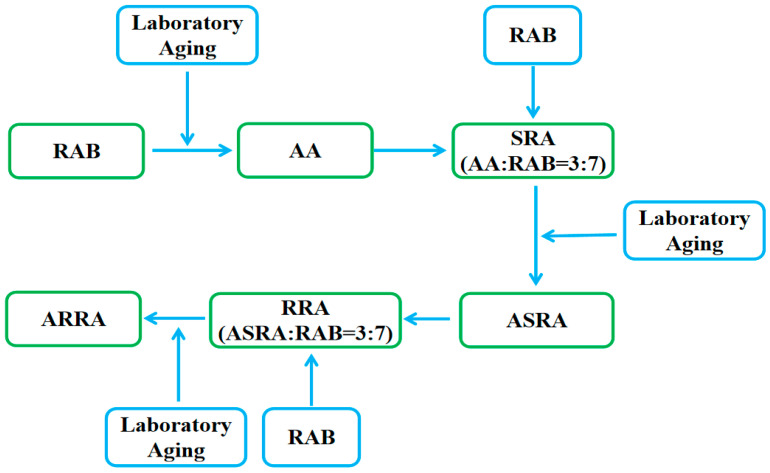
Re-recycled asphalt binder preparation process.

Penetration grade 60/80 raw asphalt binders (RAB) were used to prepare the re-recycled asphalt binders. Table 1 shows the basic indicators for RAB. In order to simulate the current production of the hot central-plant recycling, this study used 30% aged asphalt binder mixed with 70% raw asphalt binder without a rejuvenating agent to simulate recycling. The re-recycling sample preparation methods follow: (1) According to the thin-film oven test (TFOT) method in ASTM D1754, the RAB was aged at 163 °C for 5 h to simulate short-term aging. (2) After aging at 163 °C for 5 h, the TFOT RAB was placed in a pressure-aging vessel (PAV) and aged at 100 °C and 2.1 MPa for 20 h to prepare the aged asphalt (AA) for simulating long-term aging according to ASTM D6521. (3) The single recycled asphalt binder (SRA) with RAB: AA as 7:3 was prepared. (4) After preparing the SRA, the SRA used the TFOT and PAV methods to prepare the aged single recycled asphalt binder (ASRA). (5) Re-recycled asphalt binder (RRA) with RAB: ASRA as 7:3 was prepared. (6) After preparing the RRA, the RRA used the TFOT and PAV methods to prepare aged re-recycled asphalt binders (ARRA). Figure 1 shows the detailed preparation process. Table 2 shows the sample ID and corresponding description.

### 2.2. Rheological Properties Test

#### 2.2.1. Viscosity Test

The viscosity of the asphalt binder was tested at 135 and 175 °C by using a Brinell viscometer according to ASTM D4402.

#### 2.2.2. DSR Test

The DSR was used for temperature scanning of asphalt binders according to ASTM D7175. The temperature range was 46–76 °C with a 1.59 Hz frequency for G*/sinδ. Additionally, the temperature range was 16–31 °C with a 1.59 Hz frequency for G*·sinδ.

#### 2.2.3. BBR Test

The low-temperature creep properties of asphalt binders were tested at −12, −18, and −24 °C by BBR according to AASHTO T 313.

### 2.3. Microstructure Test

#### 2.3.1. AFM Test

AFM tested the microstructure of asphalt binders with a scanning range of 15 μm × 15 μm; the scanning mode was the peak force tap model. In addition, the silicon probe’s equivalent radius of curvature was 20 nm, and the surface energy was 1389.99 mJ·m^−2^. Nanoscope analysis 1.5 software was used to analyze the asphalt binder’s average microroughness (Ra) and adhesion.

#### 2.3.2. FTIR Test

The chemical functional groups of asphalt binders were characterized and quantified by the Bruker FTIR with the resolution set at 4 cm^−1^ and the scan range controlled from 400 to 4000 cm^−1^.

## 3. Results and Discussions

### 3.1. Rheological Properties Analysis

#### 3.1.1. High-Temperature Properties Analysis

**Figure 2:**
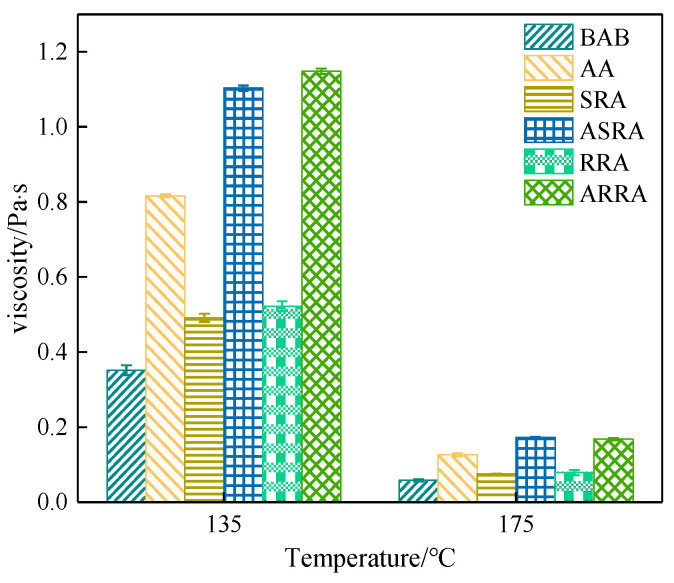
Viscosity.

Figure 2 demonstrates that the viscosity of recycled asphalt is greater than that of RAB; recycling reduces the construction ease of recycled asphalt pavement. In addition, the viscosity of RRA is greater than that of SRA at 135 and 175 °C. Re-recycling increases the viscosity of asphalt; previous studies also show that multiple recycling reduces the workability and the compactibility of recycled asphalt pavement [18].

**Figure 3:**
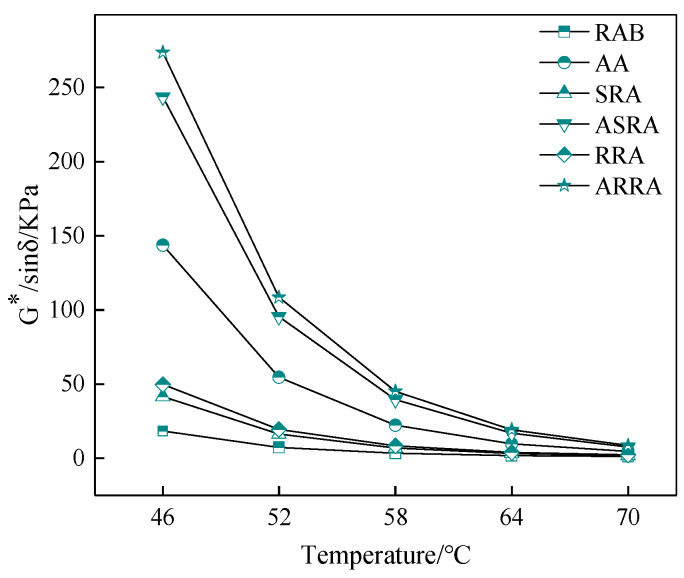
G*/sinδ.

Figure 3 demonstrates that the G*/sinδ of recycled asphalt is greater than that of RAB; recycling improved the high-temperature performance of asphalt binder. The G*/sinδ of RRA is greater than that of SRA; the re-recycling improves the high-temperature performance of asphalt. In addition, the gap in the G*/sinδ value between RRA and SRA increases after aging. It indicates that aging reduces the aging resistance of re-recycled asphalt binders from the macro performance perspective [11].

#### 3.1.2. Fatigue Performance Analysis

**Figure 4:**
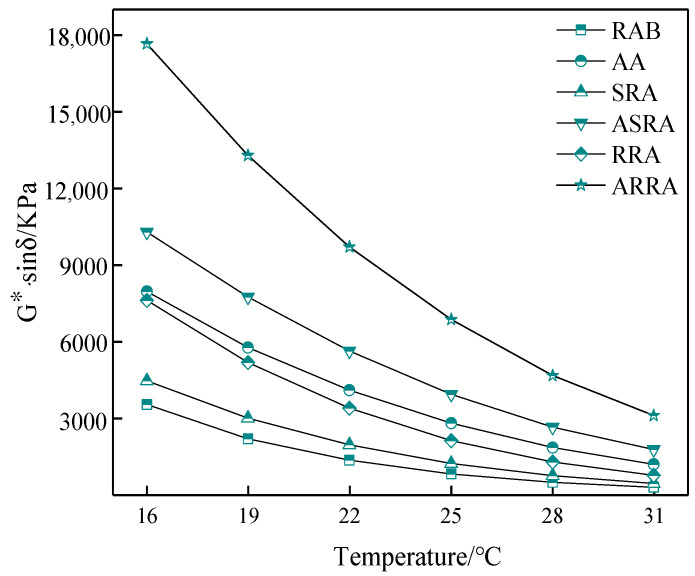
G*·sinδ.

Figure 4 shows that the G*·sinδ of recycled asphalt is greater than that of RAB; recycling significantly increases the G*·sinδ and reduces the fatigue performance of asphalt binder. The G*·sinδ of the RRA is larger than that of the SRA; the re-recycling reduces the fatigue performance of asphalt. Thus, the fatigue performance of multiple recycled asphalt should be taken seriously [19,20]. In addition, the fatigue performance gap between RRA and SRA increases after aging, indicating that the aging resistance of recycled asphalt decreases with recycling times.

#### 3.1.3. Low-Temperature Performance Analysis

**Figure 5:**
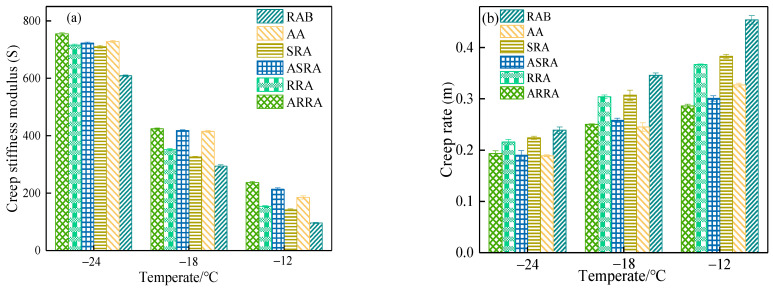
(**a**) Creep stiffness modulus and (**b**) creep rate.

Figure 5 demonstrates the S and m values of asphalt. The S value increases and the m value decreases, indicating that the low-temperature performance of asphalt decreases. Compared with the raw asphalt binder, the low-temperature performance of the recycled asphalt binder decreases. As the recycling times increase, the S value increases and the m value decreases at −12 °C. It indicates that re-recycling decreases the low-temperature performance of recycled asphalt. The S and the m value of SRA and RRA can be distinguished at −12 °C. However, before and after aging at −24 °C, the differences between SRA and RRA are small in their S and m values. Based on the results shown in Figure 5, ΔT_c_ was used to evaluate the low-temperature performance of re-recycled asphalt binders according to the findings of Kriz [11]. ΔT_c_ was calculated according to Equation (1). As ΔT_c_ decreases, it indicates a higher risk of non-load cracking of the asphalt. When ΔT_c_ ≤ −2.5 °C, the asphalt binder is prone to crack under low-temperature conditions [21].

(1) $$\Delta T_{c}{=T}_{\mathrm{cS}}-T_{\mathrm{cm}}$$

where T_cS_ is a critical low-temperature grade predicted by the S value, T_cm_ is a critical low-temperature grade predicted by the m value.

**Figure 6:**
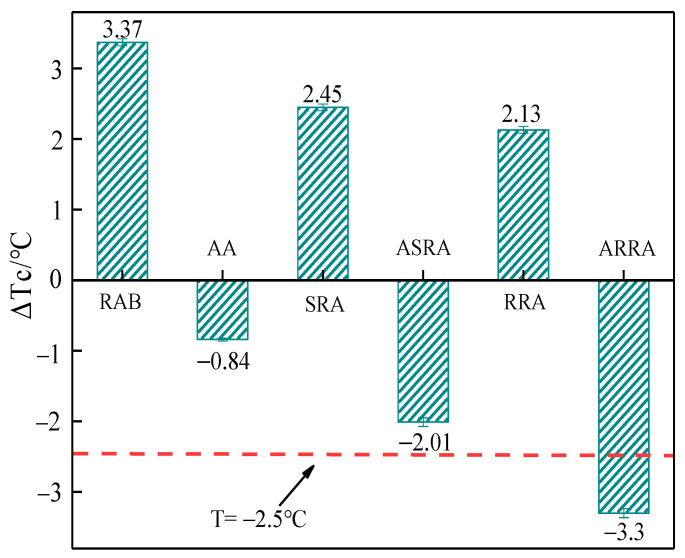
ΔT_c_.

Figure 6 demonstrates the ΔT_c_ of asphalt binders. Figure 6 shows that the differences between SRA and RRA are small in ΔT_c_. However, the ARRA ΔT_c_ ≤ −2.5 °C indicates that the ARRA will be at risk of low-temperature cracking. Therefore, ΔT_c_ can be used as an effective index to evaluate the low-temperature performance of re-recycled asphalt binders [22].

In summary, re-recycling increased the high-temperature performance of asphalt binders. However, it significantly affects fatigue and low-temperature performance, and ΔT_c_ can be used as an effective evaluation index for the low-temperature performance of re-recycled asphalt. In addition, as the recycling time increases, the aging resistance of asphalt decreases, and aging can be used as an essential condition to evaluate the properties of re-recycled asphalt. According to the assumption of Kriz [11], which does not consider the miscibility of the aged asphalt and the raw asphalt in the recycled asphalt, the SRA comprises 30% aged asphalt and 70% raw asphalt. The RRA comprises 9% secondary-aged asphalt, 21% single-aged asphalt, and 70% raw asphalt. However, due to the miscibility of aged and raw asphalt, the proportion of binder that has undergone secondary aging is less than 9% in RRA. However, this fraction of less than 9% significantly affects the rheological properties of re-recycled asphalt. Thus, the negative impact of re-recycling with 30% aged asphalt without a rejuvenating agent cannot be ignored.

### 3.2. Microstructure Analysis

#### 3.2.1. AFM Test Analysis

**Figure 7:**
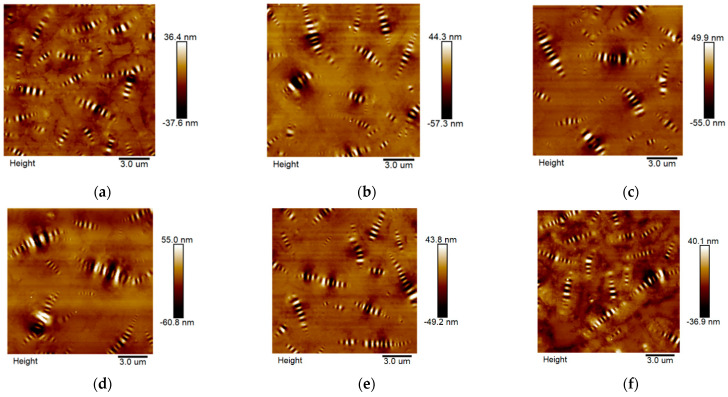
Micromorphology of asphalt binders: (**a**) RAB; (**b**) AA; (**c**) SRA; (**d**) ASRA; (**e**) RRA; (**f**) ARRA.

As shown in Figure 7, light alternating with dark elliptical features in the 2D asphalt microstructure are referred to as bee-shaped structures. The bright strip corresponds to the raised part of the asphalt microstructure, and the dark strip corresponds to the recessed part. Currently, there is no consensus on the formation of the bee-shaped structure [23]; most experts agree that it is caused by the interaction between the wax component and the matrix phase (the nonwax component of the asphalt) in the asphalt [24,25,26]. The different mechanical characteristics between wax crystal and the matrix phase lead to the formation of curvature elastic strain at their bond. To maintain constant energy in the whole system, the wax crystal counteracts the curvature elastic strain by forming a corrugated structure, which eventually forms a bee-shaped structure along the long axis [27].

The number of bee-shaped structures in the raw asphalt is large, and the single area of bee-shaped structures is small. After aging and recycling, the number of bee-shaped structures decreases, and the area of a single bee-shaped structure increases due to the reduced compatibility between the crystalline fraction and the more polar matrix phase [28]. The phenomenon above indicates that aging and recycling without a rejuvenating agent promote the further development of bee-shaped structures [17]. Furthermore, there is no significant difference between SRA and RRA in micromorphology. To further analyze the difference between SRA and RRA, a quantitative method was used to clarify the microstructure changes.

**Figure 8:**
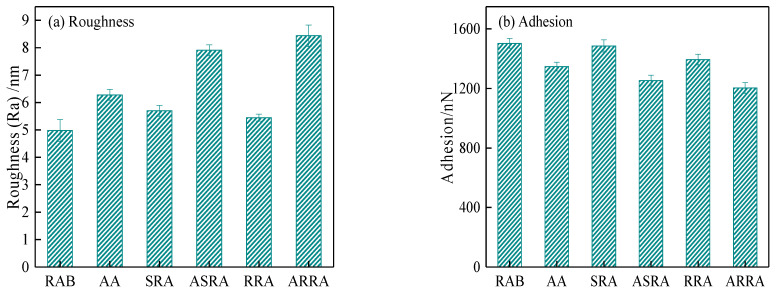

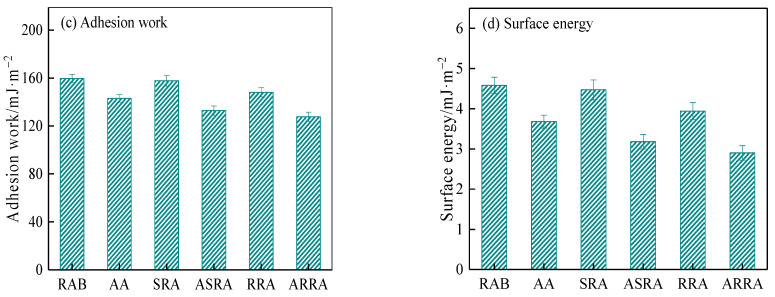
Microstructure parameters of asphalt binders.

The Johnson–Kendall–Roberts (JKR) contact mechanics model was used to calculate the adhesion work between the asphalt binder and the probe [29]. In addition, the adhesion work was converted into surface energy based on the Fowkes model [30]. The calculated results are shown in Figure 8.

As shown in Figure 8a, the aged asphalt’s roughness increases due to decreased compatibility between the wax crystal and polar matrix phase. Compared to the aged asphalt, due to the addition of 70% raw asphalt, the roughness of the recycled asphalt binder is reduced, and its surface is smoothed [31]. As the recycling times increase, the roughness in recycled asphalt changes slightly. However, the roughness of SRA and RRA increased by 38.7% and 54.8% after aging, indicating that recycled asphalt’s aging resistance decreases as the recycling times increase from the microscopic scale. This result is consistent with the conclusion above from the macroscopic performance perspective; the microstructural changes are consistent with macro performance evolution.

AFM can indirectly characterize the adhesion between asphalt binder and aggregate, as the adhesion is shown in Figure 8b. Aging reduces the adhesion between the asphalt binder and the aggregate. Recycling improves the adhesion of recycled asphalt compared to aged asphalt [32], but it is still smaller than raw asphalt. However, as the recycling times increase, the adhesion of recycled asphalt gradually decreases. Therefore, re-recycled asphalt mixtures have a greater risk of adhesion cracking than the neat asphalt mixture [18]. In addition, the evolution of adhesion work and surface energy is consistent with adhesion, as shown in Figure 8c,d.

#### 3.2.2. FTIR Test Analysis

Two aging-related indices, the sulfoxide index (I_S=O_) and the carbonyl index (I_C=O_), were calculated according to Equations (2) and (3), respectively [33,34].

(2) $$I_{S=O}=\frac{A_{1030}}{\sum A}$$

(3) $$I_{C=O}=\frac{A_{1700}}{\sum A}$$

where ΣA refers to the sum of total peak area between 2800 and 3000 cm^−1^.

**Figure 9:**
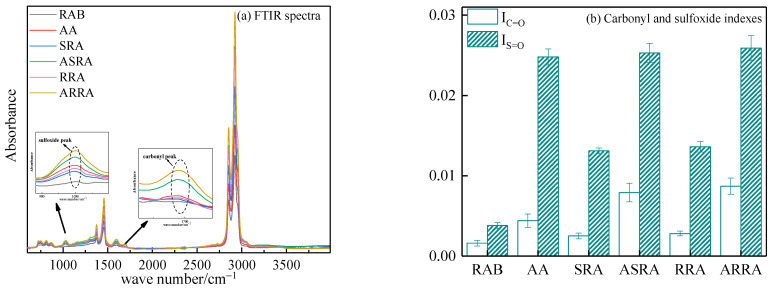
FTIR spectra of asphalt binders.

The carbonyl peak at 1700 cm^−1^ and the sulfoxide peak at 1030 cm^−1^ in FTIR spectra are often used to characterize the aging degree of asphalt binder. Figure 9 demonstrates the carbonyl and sulfoxide index. Compared to RAB, I_C=O_ of SRA and RRA increase by 56.25% and 75%, respectively, and I_S=O_ of SRA and RRA increase by 244.7% and 257.9%, respectively. Re-recycling promotes the accumulation of aged functional groups in the asphalt. Thus, re-recycling improves the high-temperature performance and reduces the fatigue and low-temperature performance of asphalt. Compared to AA, I_C=O_ of ASRA and ARRA increase by 79.55% and 97.73%, respectively. Aging promotes the accumulation of aged functional groups in the re-recycled asphalt; therefore, re-recycling reduces the anti-aging performance of asphalt. In addition, compared to AA, the I_S=O_ of ASRA and ARRA increase by 2.02% and 4.44%, respectively. The I_S=O_ between ASRA and ARRA change less; this finding is consistent with Kriz [11] and Hugener [12]. Without considering the miscibility of the aged and raw asphalt, SRA contains 30% aged and 70% raw asphalt. When SRA undergoes aging again, most of the sulfur in the 30% aged asphalt was consumed due to sulfur being more reactive than carbon [35]; only the sulfur in 70% raw asphalt generates sulfoxide, such as RRA. Therefore, the I_S=O_ between ASRA and ARRA changed less.

### 3.3. Macro–Micro Correlation

The changes in the rheological properties of asphalt are related to its microstructure [36,37]. Therefore, the rheological property parameters (viscosity at 135 °C, G*/sinδ at 64 °C, G*·sinδ at 16 °C, and ΔTc) were established to correlate with roughness (Ra), surface energy (SE), and aging index (I_Aging_= I_S=O_ + I_C=O_).

**Figure 10:**
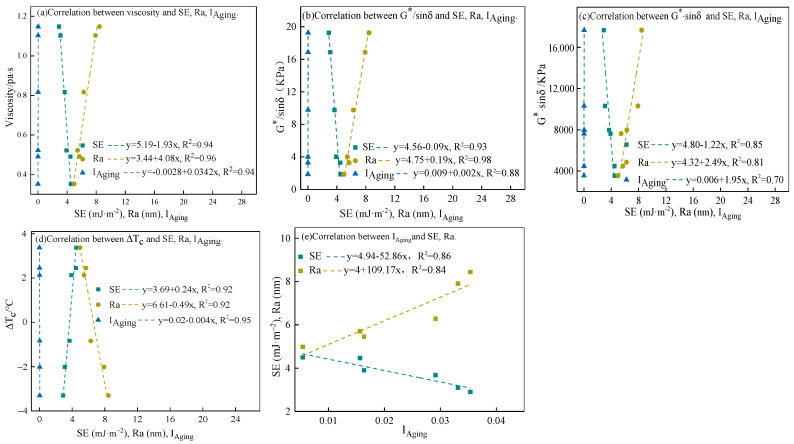
Correlation of rheological properties with microstructure.

As shown in Figure 10, except for G*·sinδ, the G*/sinδ, viscosity, and ΔT_c_ strongly correlate with Ra, SE, and I_Aging_, respectively. I_Aging_ and Ra correlate positively with viscosity, G*/sinδ, and G*·sinδ, respectively; however, I_Aging_ and Ra correlate negatively with ΔT_c_. In addition, SE correlates negatively with viscosity, G*/sinδ, and G*·sinδ, and correlates positively with ΔT_c_. Carbonyl and sulfoxide formed during the aging process influence the hardening and reduce the toughness of asphalt [38,39]. Therefore, as the I_Aging_ increases, the viscosity and high-temperature properties increase, fatigue and low-temperature properties decrease. In addition, carbonyl and sulfoxide increase the polarity of the asphalt, which decreases the surface energy of the asphalt [40,41], and the increased polarity of asphalt leads to surface roughness increase, as Figure 8a shows. In summary, I_Aging_ correlates with Ra and SE, as shown in Figure 10e, and I_Aging_ correlates with asphalt properties. Therefore, I_Aging_, Ra, and SE correlate with asphalt properties, as shown in Figure 10a–c. The microstructural changes in asphalt significantly affect the macroscopic properties.

## 4. Conclusions

1. Re-recycling improves the high-temperature performance of asphalt binder; however, it significantly reduces fatigue and low-temperature performance. The aged re-recycled asphalt’s ΔT_c_ ≤ −2.5 °C; the re-recycled asphalt has the risk of low-temperature cracking. ΔT_c_ is an effective index to evaluate the low-temperature performance of re-recycled asphalt.
2. Aging accelerates the decay of the rheological properties and significantly increases the microscopic roughness and carbonyl index of re-recycled asphalt. The macro–micro results show that re-recycling significantly reduces the anti-aging performance of asphalt.
3. As recycling times increase, the adhesion between asphalt binder and aggregate gradually decreases. Therefore, the re-recycled asphalt mixture has a greater adhesion cracking risk.
4. The carbonyl index increases significantly in recycled asphalt binders before and after aging. However, the sulfoxide index has a small change in aged recycled asphalt binders because of sulfur consumption.
5. The macroscopic properties are strongly correlated with aging functional groups, roughness, and surface energy of recycled asphalt; aging functional groups also strongly correlate with surface energy and roughness. The microstructural changes in asphalt significantly affect the macroscopic properties.
6. All results are based on asphalt experiments; however, the asphalt mixture is also an essential part of recycling, and additional asphalt mixture experiments are crucial. In addition, multiple recycled asphalt preparation method is complex; a simple preparation method is urgently needed.

## Figures and Tables

**Table 1:** Basic index of raw asphalt binder.

Technical Index	Results
Penetration Value/0.1 mm	77.76
Softening Point/°C	47.85
Ductility at 10 °C/cm	>100

**Table 2:** Sample ID and corresponding description.

Number	Sample ID	Description
1	TFOT	Thin-film oven test
2	PAV	Pressure aging vessel
3	RAB	Raw asphalt binder
4	AA	Aged raw asphalt binder
5	SRA	Single recycled asphalt binder
6	ASRA	Aged single recycled asphalt binder
7	RRA	Re-recycled asphalt binder
8	ARRA	Aged re-recycled asphalt binder
